# Using indirect calorimetry in place of fixed energy prescription was feasible and energy targets were more closely met: do not forget an important limitation

**DOI:** 10.1186/s13054-020-03075-2

**Published:** 2020-06-19

**Authors:** Patrick M. Honore, Leonel Barreto Gutierrez, Luc Kugener, Sebastien Redant, Rachid Attou, Andrea Gallerani, David De Bels

**Affiliations:** ICU Department, Centre Hospitalier Universitaire Brugmann, Place Van Gehuchtenplein, 4, 1020 Brussels, Belgium

We read with great interest the recent article by Lambell et al. discussing nutrition therapy in critically ill patients and the role of indirect calorimetry (IC) [1]. Indirect calorimetry allows for the measurement of VO2 and VCO2 through the ventilator and is the gold standard method for measuring resting energy expenditure (REE) in critical illness when ideal test conditions are implemented [1]. Both the European (ESPEN) and American (ASPEN/SCCM) clinical practice guidelines recommend the use of IC to measure energy expenditure [1]. At this time, there are only three randomized controlled trials (RCTs) comparing IC with formulae (25 kcal/kg/day) [1]. In all three RCTs, indirect calorimetry was feasible and energy targets were more closely met when using IC in place of fixed energy prescription [1]. While supporting the use of IC in some settings, we believe it is important to warn clinicians about a limitation of the technique, particularly when patients are under continuous renal replacement therapy (CRRT) [2]. Fifty percent of the critically ill septic and non-septic population develop acute kidney injury, and 25% require renal replacement therapy (RRT) [3]. Patients undergoing CRRT lose a substantial amount of CO^2^, in gas form and as bicarbonate, in the effluent, making IC unreliable [4]. This is also true for IC performed in patients receiving extracorporeal membrane oxygen (ECMO), unless a mathematical correction is applied [5]. It is important that clinicians are aware not only of the indications of IC, but also of the limitations.

## Authors’ response

### Response to the letter to the editor: “Using indirect calorimetry in place of fixed energy prescription was feasible and energy targets were more closely met: do not forget an important limitation”

Kate J Lambell, Oana A Tatucu-Babet and Emma J Ridley

We thank Professor Honore and colleagues for their interest in our paper *“Nutrition therapy in critically illness: a review of the literature for clinicians”* [1]. In the review, we discuss the clinical guideline recommendations and evidence supporting the use of indirect calorimetry (IC) to measure energy expenditure and guide energy delivery [1]. We highlight that energy targets are met more closely with the use of IC than predictive equations, but there are limited studies reporting a benefit on clinical outcomes when IC is used. To further investigate the impact of using IC on clinical outcomes, we recently published a systematic review, evaluating if energy delivery guided by IC impacted hospital mortality and other important outcomes compared to when predictive equations were used [6]. We identified four randomized control trials and found no differences in intensive care unit mortality and hospital length of stay between groups [6]. However, the duration of mechanical ventilation was increased when IC guided energy delivery [6]. Further investigation is required to understand how the use of IC to guide energy delivery impacts clinical and functional outcomes in critically ill adults, particularly across different phases of illness. And, as pointed out by Professor Honore and colleagues, there are limitations in some populations.

Importantly, Professor Honore and colleagues highlight that the use of IC may not be reliable during continuous renal replacement therapy (CRRT) andextracorporeal membrane oxygenation (ECMO) due to the removal of CO_2_ by the membrane in CRRT and inability to capture O_2_ uptake and CO_2_ removal during ECMO. To ensure accurate determination of energy expenditure, all limitations to IC must be considered, and we refer readers to a comprehensive review outlining technical factors affecting IC measurement [7]. Of interest, investigation to try and understand the influence of CRRT on VCO_2_, VO_2_, and energy expenditure (by IC) has recently been published [8]. In a small observational study of 10 critically ill patients receiving CRRT, CO_2_ removal by CRRT led to a minimal change of 3% in measured energy expenditure, a difference that is not considered clinically important [8]. In addition to the study mentioned by the authors, another study has been completed [9], and another is underway to develop methods to accurately measure energy expenditure in patients receiving ECMO (*ACTRN12619000760178*). These are important studies investigating methods to measure energy expenditure using IC in nutritionally vulnerable populations where limitations to traditional IC exist.

## Data Availability

Not applicable.

## Abbreviations

IC: Indirect calorimetry
REE: Resting energy expenditure
RCTs: Randomized controlled trials
RRT: Renal replacement therapy
CRRT: Continuous renal replacement therapy
